# Simultaneous Endocardial and Epicardial Approach for Successful Closure of Left Atrial Appendage With Difficult Anatomy

**DOI:** 10.1016/j.jaccas.2025.104338

**Published:** 2025-07-30

**Authors:** Aashish Katapadi, Rakesh Gopinathannair, Naga Venkata K. Pothineni, Douglas Darden, Hemant Godara, Dhanunjaya Lakkireddy, Rajesh Kabra

**Affiliations:** a Kansas City Heart Rhythm Institute, Overland Park, Kansas, USA; b University of Missouri, Columbia, Missouri, USA

**Keywords:** atrial fibrillation, computed tomography, occlude, stroke

## Abstract

**Background:**

Left atrial appendage closure (LAAC) is increasingly used for stroke prevention; however, left atrial appendage (LAA) anatomy may pose significant challenges.

**Case Summary:**

A 67-year-old man with paroxysmal atrial fibrillation, CHA_2_DS_2_-VASc Score of 3, and recurrent falls was referred for LAAC. Owing to challenging anatomy noted during preoperative imaging, epicardial access was obtained at procedure onset. Following multiple unsuccessful endocardial attempts, LAAC was successfully achieved by an epicardial device.

**Discussion:**

Preprocedural imaging can identify challenging LAA anatomy and assist in procedural planning. In patients with challenging LAA anatomy, obtaining epicardial access up front before endocardial closure attempts offers an additional immediate option in case of failure. This case highlights the importance and use of both endocardial and epicardial LAAC strategies for a comprehensive LAAC program.

**Take-Home Message:**

We demonstrate that a methodological approach with preprocedural LAA imaging and endocardial and epicardial LAAC strategies results in successful clinical outcomes.

## Visual Summary

**Figure:**
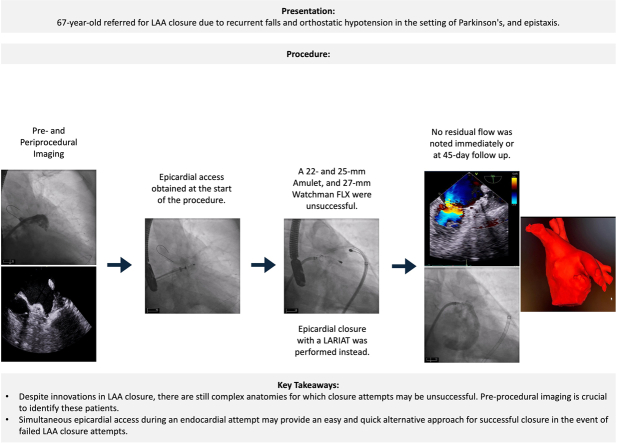

## History of Presentation

A 67-year-old man with a history of paroxysmal atrial fibrillation (AF) with a CHA_2_DS_2_-VASc Score of 3 and a history of recurrent falls was referred for left atrial appendage closure (LAAC) following prior unsuccessful attempts at endocardial closure with a Watchman device (Boston Scientific) at another center.

**Take-Home Messages**

• Despite innovations in LAAC, there are still complex LAAs for which closure attempts may be unsuccessful. Preoperative imaging with cardiac CT angiography and/or TEE is crucial to identify these patients.
• Simultaneous epicardial access during an endocardial LAAC attempt, especially in patients with a difficult-to-close LAA, may provide an alternative approach for successful closure.

## Past Medical History

Besides AF, the patient had a history of Parkinson's disease associated with imbalance and recurrent falls.

## Differential Diagnosis

There were no applicable differential diagnoses.

## Investigations

**Figure 1:**
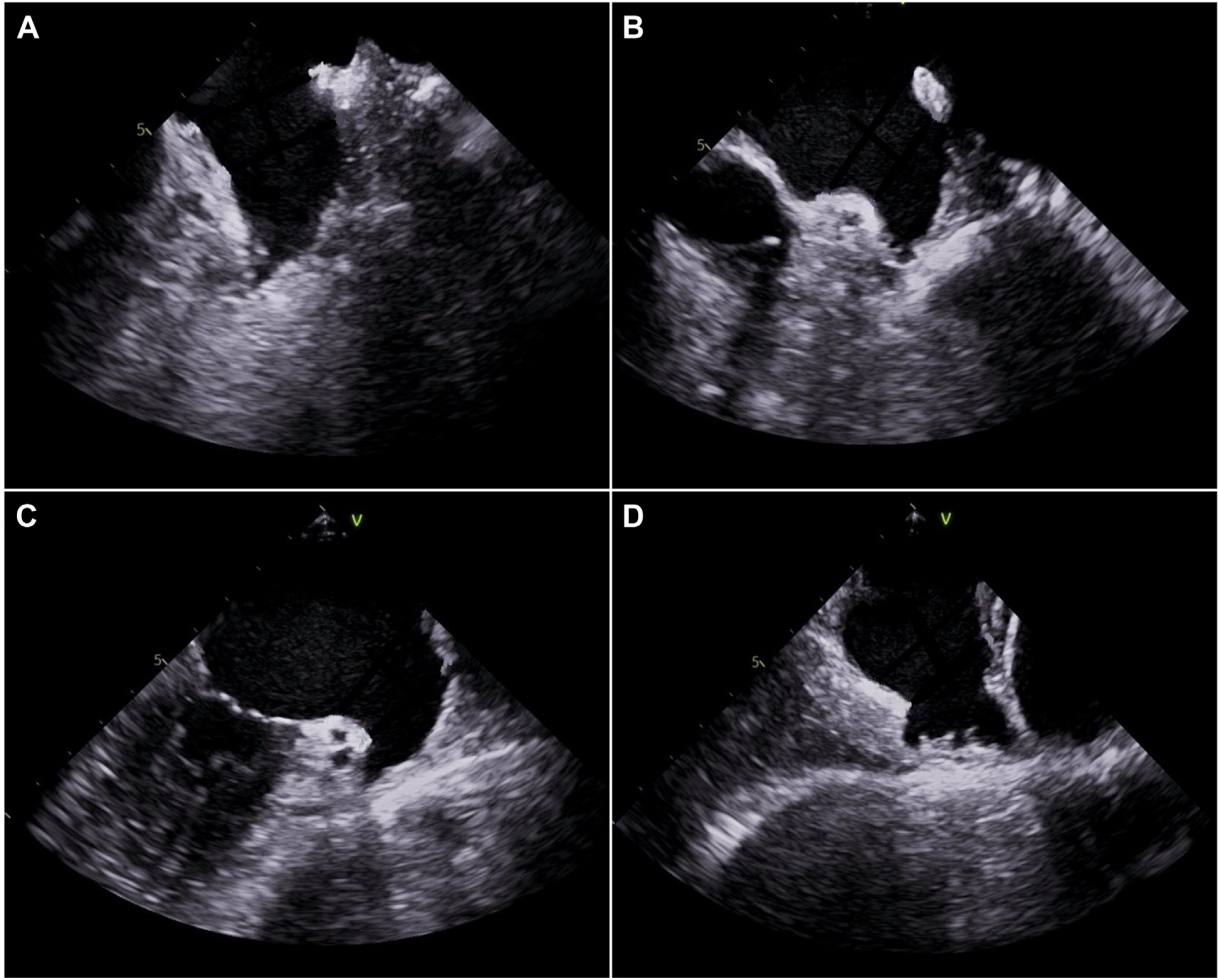
LAA Transesophageal Echocardiogram The left atrial appendage was visualized in the 0° (A), 45° (B), 90° (C), and 135° (D) views. The maximal left atrial appendage (LAA) ostium width and depth were 24 mm and 12 mm, respectively.

Preoperative transthoracic echocardiography and transesophageal echocardiography (TEE) were performed. Transthoracic echocardiography showed a left ventricular ejection fraction of 66%, mildly dilated left atrium, and mild mitral regurgitation. TEE showed a maximal left atrial appendage (LAA) ostium width of 24 mm and maximal LAA depth of 12 mm (Figures 1A to 1D).

## Management

**Figure 2:**
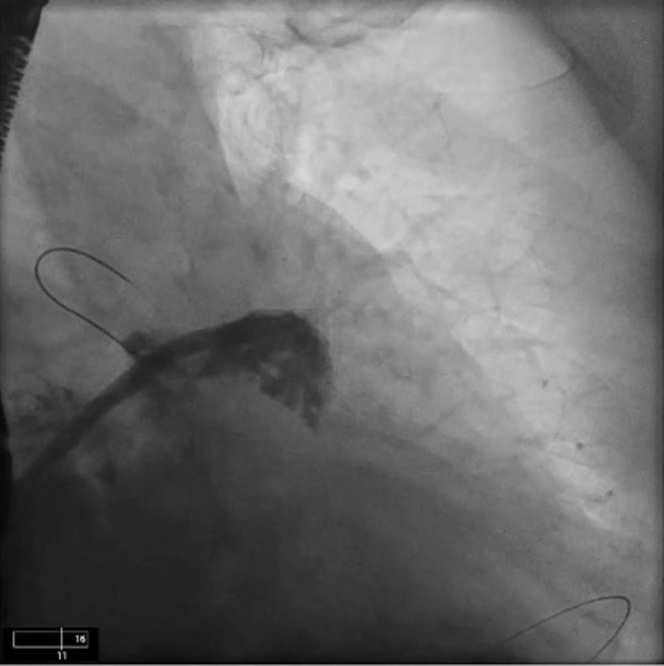
Left Atrial Appendogram in Right Anterior Oblique Caudal View The appendogram revealed a small anterior lobe with significant distal trabeculations. A guidewire in pericardial space is also seen.

**Figure 3:**
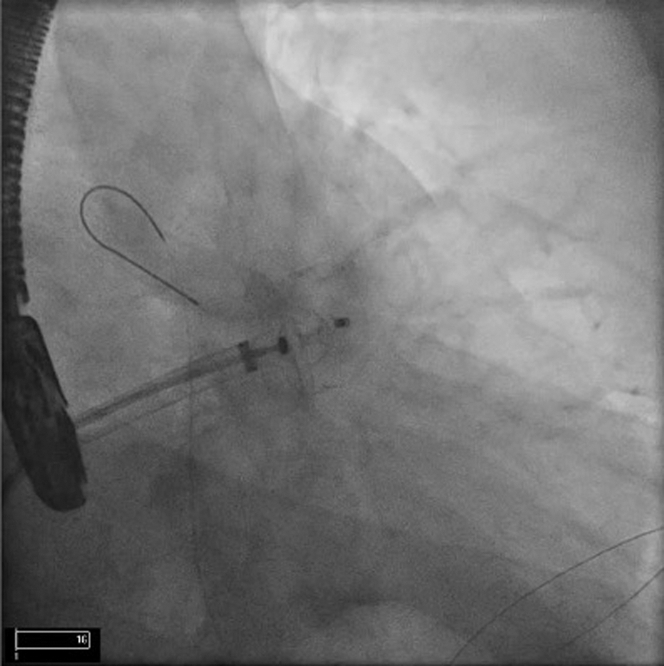
Amulet Device Tug Test The Amulet device dislodged out of the left atrial appendage during the tug test. The second attempt at a larger device also failed the tug test.

**Figure 4:**
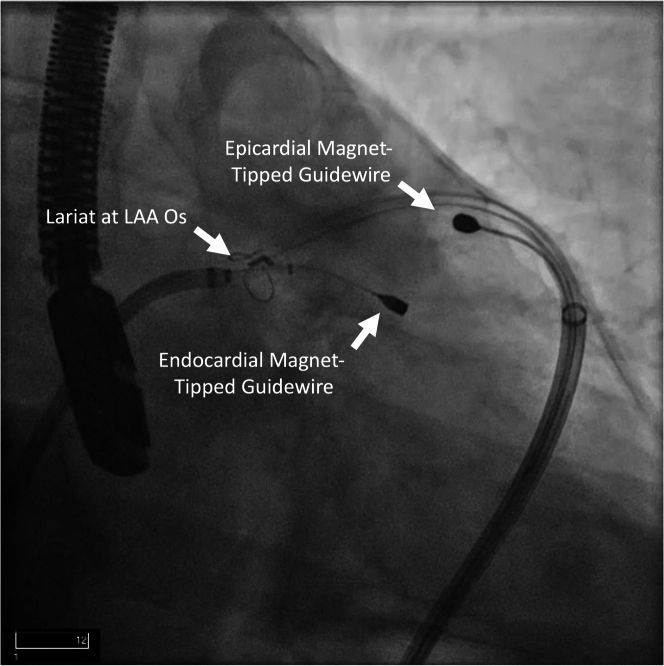
Epicardial Left Atrial Appendage Closure Exclusion With Lariat in Right Anterior Oblique Caudal View Using the endocardial and epicardial magnet-tipped guidewires as a rail, the Lariat was successfully deployed epicardially at left atrial appendage os. LAA = left atrial appendage.

**Figure 5:**
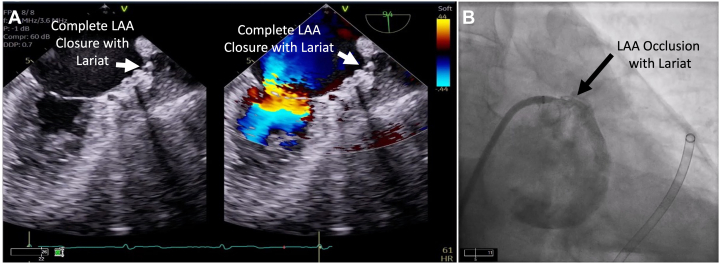
Postoperative Confirmation of Left Atrial Appendage Occlusion Complete closure of the left atrial appendage without any residual flow across the ostium was noted with transesophageal echocardiography (A) and left atrial angiography (right anterior oblique caudal view) (B). Abbreviation as in Figure 4.

There were concerns regarding the relatively wide LAA ostium, limited depth, and distal LAA trabeculations on TEE, potentially making endocardial LAAC challenging. Hence, it was decided to obtain empirical epicardial access for the potential use of an epicardial LARIAT device (Atricure) if the endocardial LAAC failed (Table 1). The procedure was performed under general anesthesia with TEE performed by a cardiologist. Under fluoroscopic guidance, epicardial access was obtained with a micropuncture needle, and a guidewire was placed into the epicardial space. Subsequently, ultrasound-guided right femoral venous access was obtained. The patient was administered heparin, and a transseptal puncture was performed to access the LAA. A left atrial appendogram was obtained, which demonstrated a small anterior lobe with significant trabeculations (Figure 2, Video 1). A 22-mm Amulet (Abbott Cardiovascular) device was prepared, advanced into the LAA through the transseptal sheath, and deployed under fluoroscopic and TEE guidance. However, during the tug test, the device dislodged out of LAA (Figure 3, Video 2). The device was fully recaptured and was replaced by a 25-mm Amulet device, which also dislodged during the tug test; it was fully recaptured and removed. An alternative endocardial LAAC device—the Watchman FLX (Boston Scientific)—was attempted. A 27-mm Watchman FLX device was deployed but failed the tug test. Lastly, a 31-mm Watchman was attempted but noted to have significant shoulders and could not be deployed any deeper due to limited depth and distal trabeculations of the LAA. The device was fully recaptured and removed. As multiple endocardial LAAC devices were unsuccessful, it was decided to proceed with epicardial LAAC with the LARIAT device. A magnet-tipped guidewire was inserted into the LAA through the transseptal sheath. Next, the micropuncture guidewire in the pericardial space was exchanged for a stiffer guidewire over which an epicardial sheath and a second, magnet-tipped guidewire was advanced and attached to the endocardial magnet. Using the magnet-tipped guidewires as a rail, a LARIAT device was deployed epicardially to exclude the LAA under fluoroscopic and TEE guidance (Figure 4). Complete closure of the LAA without any residual flow across the ostium was noted with both TEE and left atrial angiogram (Figures 5A and 5B, Videos 3 and 4). A pericardial drain was placed at the end of the procedure.

**Table 1:** Equipment List for Endocardial/Epicardial Approach

Equipment	Manufacturer
LAA closure and device delivery	
22- and 25-mm Amulet	Abbott Cardiovascular
TorqVue Delivery System	Abbott Cardiovascular
27-mm Watchman FLX	Boston Scientific
45-mm LARIAT Suture Delivery Device	Atricure
EndoCath Occlusion Balloon	Atricure
FindrWirz Magnetic Guidewires	Atricure
SureCUT Suture Cutter	AtriCure
TenSURE Suture Tightener Device	AtriCure
Pericardial drainage	
Perivac pericardiocentesis kit	Boston Scientific
Imaging	
Vivid ultrasound	GE Healthcare
EPIQ CVx transesophageal echocardiogram	Philips Healthcare
Soundstar intracardiac echocardiogram	Biosense Webster
Trans-septal puncture	
NRG large curve C1 transseptal needle	Baylis Medical
5-F pigtail	Cordis
SL-1 sheath	Abbott Laboratories
SR0 guiding catheter	Abbott Laboratories
Femoral access	
Micropuncture kit	Merit Medical Systems
Amplatz Super Stiff Guidewire (Boston Scientific)	
Pinnacle arterial introducer sheath	Terumo Medical
Fast-Cath Hemostasis Introducer	Abbott Vascular
13-F soft tip cannula	Atricure
Subxiphoid access	
Galt needle	Theragenics
8-, 10-, and 12-F dilator sheaths	Cook Medical

LAA = left atrial appendage.

## Outcome and Follow-Up

**Figure 6:**
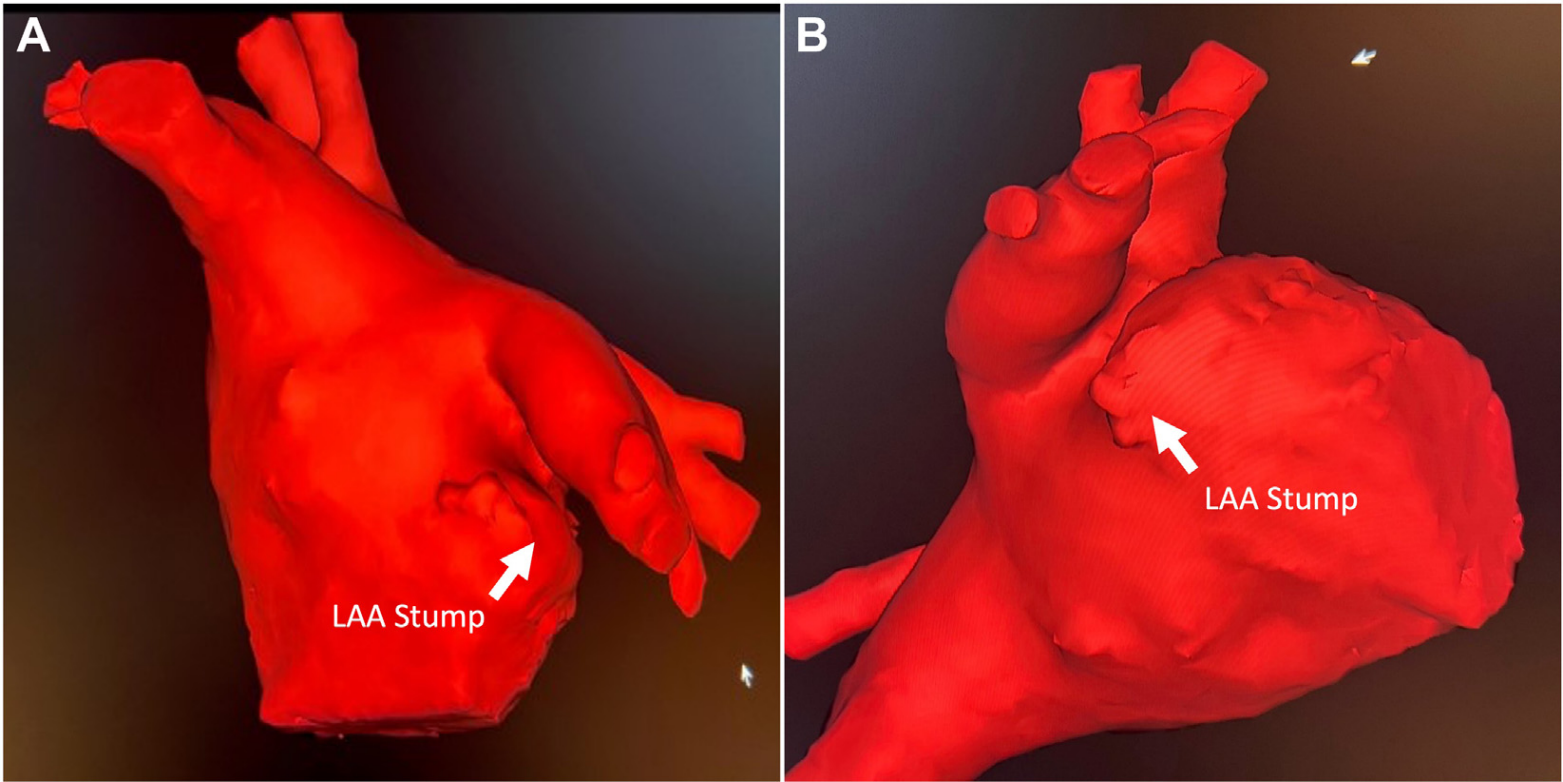
Postoperative Left Atrial Appendage Computed Tomography Images from 45-day postoperative cardiac computed tomography (A and B) reveal no residual leak, thrombus, or significant stump. Abbreviation as in Figure 4.

The patient tolerated the procedure well, and no complications were noted. The pericardial drain was removed the next day, and the patient was discharged home 2 days later. A follow-up 45-day cardiac computed tomography (CT) demonstrated persistent LAA occlusion with no inflow of intravenous dye into the LAA (Figures 6A and 6B).

## Discussion

Advances in the design of endocardial LAAC devices have significantly improved outcomes in patients with challenging LAA anatomy.^1^^,^^2^ However, endocardial LAAC may still be challenging in certain LAAs, especially those with wide os, inadequate depth, and significant trabeculations. Preprocedural imaging with cardiac CT or TEE is essential to identify these difficult anatomies. We propose empirical epicardial access in these challenging endocardial LAAC cases so that the option of epicardial LAAC with a LARIAT device can potentially be used in the same setting in case deployment of an endocardial LAAC fails.^3^ Although epicardial access may also be achieved following failed endocardial attempts during the same procedure, reversal of anticoagulation for epicardial access may increase the risk of thromboembolism, especially in the presence of a large sheath in the left atrium. Another option might be referring these patients for surgical LAA clip. However, this approach would require an additional surgical procedure that is more invasive. Our patient had a successful epicardial LAAC during the same percutaneous procedure when multiple endocardial devices failed.

Although LAAC with a LARIAT device was used as a bailout complementary strategy in our patient, it can be used as a primary strategy for patients with AF and suspected LAA arrhythmogenic triggers in an enlarged left atrium or long-standing persistent AF.^4^ Hence, an advanced LAAC center of excellence may benefit from having expertise in both endocardial and epicardial strategies.

## Conclusions

A comprehensive approach with both endocardial and epicardial options may improve the success rate for LAAC, especially in advanced LAAC centers of excellence. Empirical epicardial access should be considered during endocardial LAAC in patients with challenging LAA anatomy, such as wide os, inadequate depth, and significant trabeculations.

**Visual Summary:**
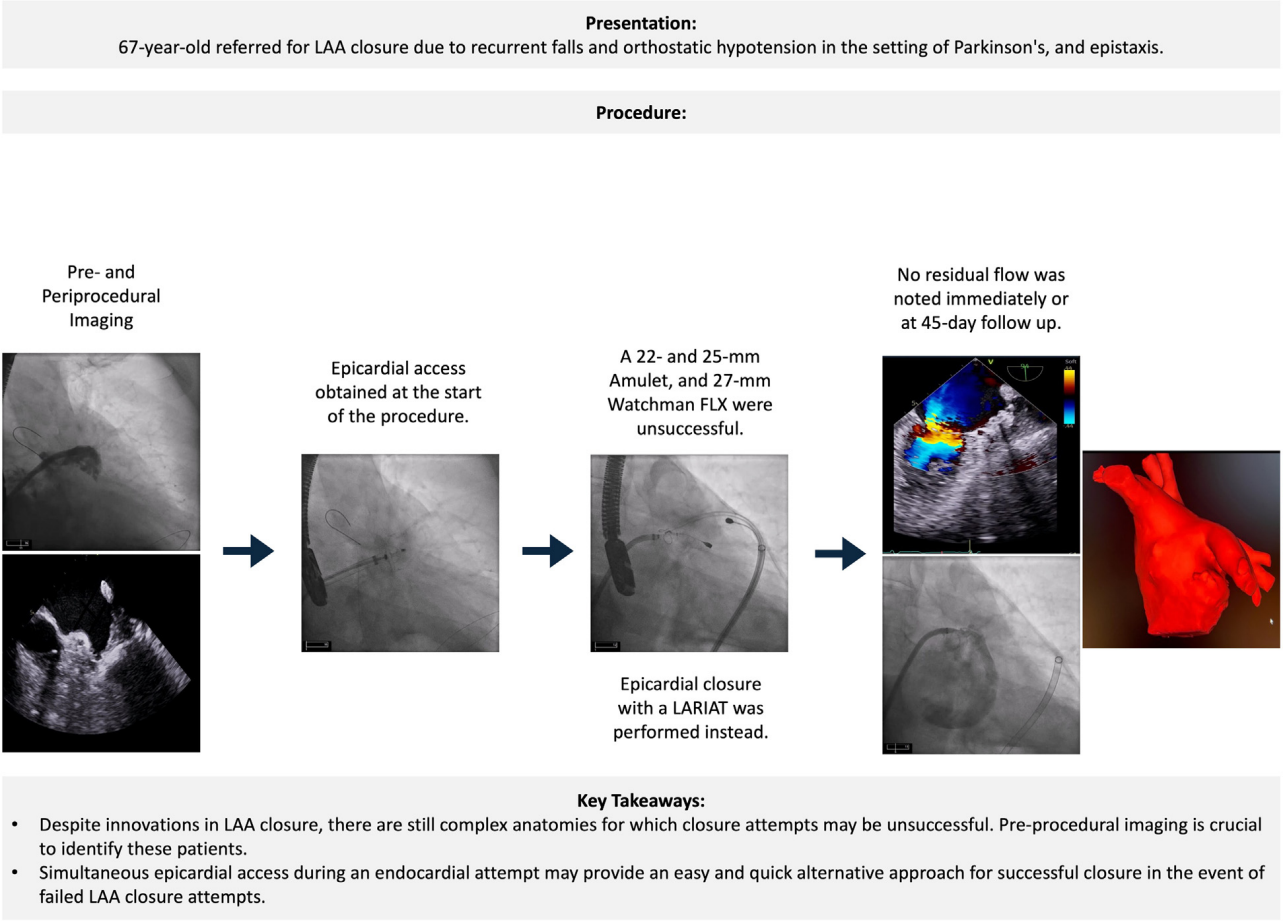
Pre-Procedural Planning and Endo-/Epicardial Approach to LAAO

## Funding Support and Author Disclosures

Dr Gopinathannair has been a consultant to Abbott Medical, a speaker for Sanofi, and on the Medical Advisory Board for Pacemate. Dr Pothineni has been a consultant to Boston Scientific. Dr Lakkireddy has been a consultant to Abbott, Acutus, AltaThera, Boston Scientific, Biosense Webster, and Medtronic. All other authors have reported that they have no relationships relevant to the contents of this paper to disclose.

## Appendix

Video 1 Left Atrial Appendogram The full appendogram of the left atrial appendage demonstrates the anterior lobe with distal trabeculations. Notably, it also shows delayed contrast flow out of the appendage, highlighting the significance of trabeculations.

Video 2 . Amulet Device Tug Test and Dislodgment Fluoroscopy revealed that the Amulet device was not positioned properly, as it failed the post-implant tug test.

Video 3 . Transesophageal Echocardiogram of Left Atrial Appendage A postoperative transesophageal echocardiogram reveals a well-excluded left atrial appendage with no significant leakage or stump.

Video 4 . Angiogram of Left Atrial Appendage The full angiogram reveals no contrast flow, again with no leak, into the left atrial appendage.
